# Improving care for residents in long term care facilities experiencing an acute change in health status

**DOI:** 10.1186/s12913-020-05919-7

**Published:** 2020-11-25

**Authors:** Abraham Munene, Eddy Lang, Vivian Ewa, Heather Hair, Greta Cummings, Patrick McLane, Eldon Spackman, Peter Faris, Nancy Zuzic, Patrick B. Quail, Marian George, Anne Heinemeyer, Daniel Grigat, Mark McMillen, Shawna Reid, Jayna Holroyd-Leduc

**Affiliations:** 1 Cumming School of Medicine, University of Calgary, University of Calgary South Tower 1403, 29th Street NW, Calgary, AB T2N 2T9 Canada; 2 Alberta Health Services, Calgary, AB Canada; 3 Faculty of Nursing, University of Alberta, Edmonton, AB Canada; 4 Resident Family Advisory Council, AHS Emergency Strategic Clinical Network, Calgary, AB Canada; 5 Department of Emergency Medicine, University of Alberta, Edmonton, AB Canada

**Keywords:** Long term care, Mixed methods, Quality of care, Geriatrics, Long-term care, Emergency departments, Community Paramedicine

## Abstract

**Background:**

Long term care (LTC) facilities provide health services and assist residents with daily care. At times residents may require transfer to emergency departments (ED), depending on the severity of their change in health status, their goals of care, and the ability of the facility to care for medically unstable residents. However, many transfers from LTC to ED are unnecessary, and expose residents to discontinuity in care and iatrogenic harms. This knowledge translation project aims to implement a standardized LTC-ED care and referral pathway for LTC facilities seeking transfer to ED, which optimizes the use of resources both within the LTC facility and surrounding community.

**Methods/design:**

We will use a quasi-experimental randomized stepped-wedge design in the implementation and evaluation of the pathway within the Calgary zone of Alberta Health Services (AHS), Canada. Specifically, the intervention will be implemented in 38 LTC facilities. The intervention will involve a standardized LTC-ED care and referral pathway, along with targeted INTERACT® tools. The implementation strategies will be adapted to the local context of each facility and to address potential implementation barriers identified through a staff completed barriers assessment tool. The evaluation will use a mixed-methods approach. The primary outcome will be any change in the rate of transfers to ED from LTC facilities adjusted by resident-days. Secondary outcomes will include a post-implementation qualitative assessment of the pathway. Comparative cost-analysis will be undertaken from the perspective of publicly funded health care.

**Discussion:**

This study will integrate current resources in the LTC-ED pathway in a manner that will better coordinate and optimize the care for LTC residents experiencing an acute change in health status.

**Supplementary Information:**

The online version contains supplementary material available at 10.1186/s12913-020-05919-7.

## Background

With more than five million Canadians over the age of 65, the demand for Long Term Care (LTC) facilities is projected to increase [1]. Currently, 7 % of seniors aged 65 and older reside in LTC or residences for senior citizens [2]. LTC facilities provide health services, personal care, and cater to the daily living needs of residents requiring assistance with care [3]. Most residents of LTC facilities are frail with end-stage chronic diseases, and therefore are vulnerable to conditions such as fall related injuries, pneumonia, urinary tract infections, congestive heart failure, and other conditions that may require medical care [4]. For some residents, transfer to emergency departments (ED) may be required, depending on the severity of the condition and the goals of care of the resident. However, many transfers from LTC to the ED are unnecessary and can increase the demand placed on ambulance transport services and ED resources. Additionally, LTC residents transferred from LTC to ED can experience discontinuity in care, prolonged wait times in the ED and exposure to iatrogenic harms, all of which can result in cognitive and functional decline [5, 6]. Furthermore, with the current coronavirus pandemic reducing transfers to the ED from LTC facilities may reduce the strain on the current health care system and reduce the risk of exposure of vulnerable seniors to the virus upon transfer.

When a transfer to the ED is indicated, there is often poor and inconsistent communication between LTC and ED staff. The literature suggests that the two main issues with transfers between LTC and the ED are related to the fact that the ED does not ask for specific information from LTC facilities, along with limited motivation and ability of LTC to support additional communication processes [7]. This can lead to a lack of understanding of a patient’s baseline functional and cognitive abilities, poor problem description and patient management, inefficient resource utilization, and patient and family dissatisfaction in the transfer process, all contributing to poor patient outcomes [8, 9]. Patient centered care (PCC) therefore becomes fundamental in achieving better outcomes for LTC residents. PCC should incorporate the patients’ reason for seeing the health practitioner, their requirement for information that pertains to their health, a holistic understanding of the patients’ life circumstances that considers their physical, emotional, and social context. Furthermore, PCC seeks to find common definitions of the patients’ health problem and management strategies that are tailored to the patients’ needs. PCC should also increase compliance to disease prevention and health promotion by the patient, and promote a mutually beneficial relationship between the patient and the health practitioner [10, 11].

Evaluating and managing acute conditions at LTC facilities may mitigate against the negative impact transfers have on LTC residents and their families, as well as better optimize health care resources. A recent study found that residents and their families were very satisfied with receiving acute care within LTC facilities through community paramedicine interventions, thus avoiding transfers to ED, as they felt that residents could be better cared for at LTC facilities [12]. Given the patient and health system challenges associated with LTC to ED transfers, seeking innovative ways to streamline the transfer process may help improve patient outcomes and decrease the system inefficiencies currently associated with transfers.

### Aim and objective

The aim of this study is to improve the care provided to LTC residents who develop an acute change in health status, using a patient-centered approach that requires the patients’ participation, involvement, enhances the relationship between the patient and health practitioners, and provides treatment in the most appropriate location [11]. We aim to optimize, standardize, and evaluate the processes followed when considering transfer of residents from LTC to an ED. Specifically, the objective of this research is to implement and evaluate an evidence-informed standardized care pathway for the care of LTC residents experiencing an acute change in health status**.**

## Methods

### Study design

**Fig. 1:**
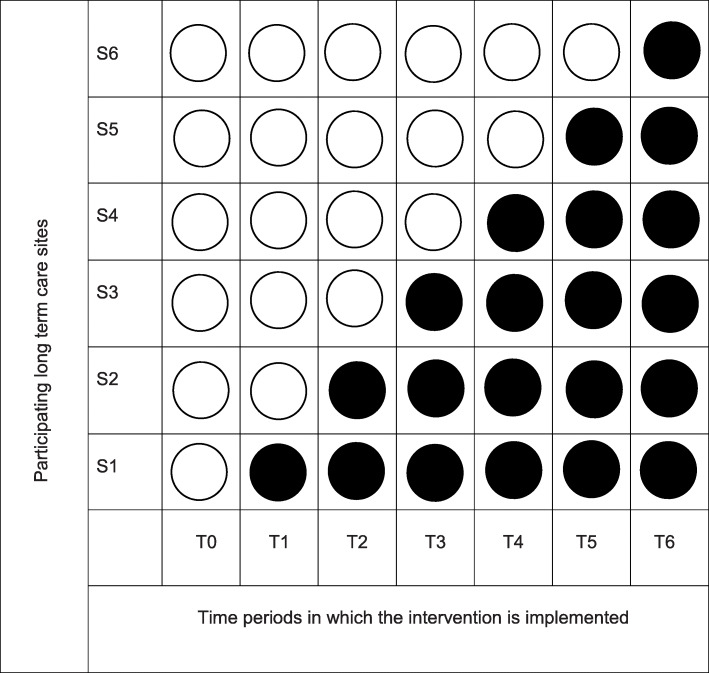
Implementation of a stepped wedge design

This is an implementation trial which will follow a Hybrid III strategy in which we are testing an implementation intervention while observing and gathering information on the clinical intervention and related outcomes. A Hybrid III strategy is useful when there is face validity for the implementation interventions that would likely be generalizable to a new setting; there is a low risk of the implemented interventions negatively impacting current practices; there is endorsement or actual mandates to the adoption of the intervention; the implementation intervention should be testable in the context of the study [13]. We will use a quasi-experimental randomized stepped-wedge design in the implementation and evaluation of the pathway (i.e., intervention) within the Alberta Health Services (AHS) Calgary zone, Alberta, Canada (Fig. 1).

By the end of the study, all 38 participating LTC facilities will have received the intervention. However, the order in which facilities receive the intervention will be determined at random [14]. A stepped-wedge design is an ideal design to evaluate an intervention on a population level, where there are implementation resource constraints. This design is also useful where there are ethical or community concerns that all participating sites should eventually get the intervention. As a crossover design, it also controls for confounding and site-level differences because each site is observed before and after the intervention. Therefore, changes within the wider health system over time can be observed, given data is available from multiple pre-intervention and intervention sites at various points in time [15].

The intervention will be implemented at four to five LTC facilities each quarter (every 3 months), with all facilities having implemented the intervention in 30 months. The pre-implementation phase will take place over 1 month. During the pre-implementation phase the researchers contact the participating sites, meet with site leaders and staff, conduct a barriers to implementation assessment, provide the overarching rationale for the implementation program and outline the plan for implementation (i.e., setting an implementation workshop and determining what additional resources are required). Baseline data with the OPTIC tool and chart abstractions will be done prior to the intervention, with implementation occurring over the subsequent 2 months. The intervention will then become an expected standard care practice at the facility, and thus this will be considered the post-implementation phase.

The order of implementation will be randomized using random number generation to reduce bias, where the unit of randomization is the LTC facility. LTC facilities with the same owner-operator will be randomized to the same implementation period, given that these facilities often share staff. Until the pathway is implemented at a LTC facility, facilities will act as controls for the purposes of analysis.

### Implementation process and strategy

This is an integrated knowledge translation project informed by the knowledge to action (KTA) cycle [16]. Specifically, we identified an evidence-informed solution to address a care gap and have engaged with relevant stakeholders. We will implement this change initiative, considering both local LTC context and barriers to implementation. The facility implementation can be modified to address these identified barriers, without altering the pathway (i.e., intervention) or the outcomes analyzed. All LTC facilities involved in the project will receive the same package and training. As to how each facility adopts the intervention is based on the discretion of the facility with the implementation team working to support and tailor implementation to each facilities’ needs and help overcome barriers identified in each facilities’ barrier assessment.

A survey to assess barriers will be completed by the staff within each LTC facility during the pre-implementation phase. By identifying potential local barriers to implementation up front, each facility can develop a local package of implementation strategies that addresses their unique implementation barriers. This barrier survey was developed using the Theoretical Domains Framework (TDF) [17–19] and successfully piloted in two continuing care facilities in Calgary. The Behaviour Change Wheel [17] which includes nine intervention elements that can be mapped to the identified barriers among 14 behaviour change domains within the TDF, will be used to help guide the implementation strategies within each LTC facility. Although the exact implementation strategies will be adapted for the local context within each facility, the implementation will include: i) education for LTC health care aids (HCAs) and nursing staff; ii) notifications sent to attending physicians in LTC; iii) site champions; iv) use of printed materials developed with support of the resident and family project team members and; v) focus on informing LTC residents and their families of this change in care processes.

A central implementation coach will provide support to the LTC staff to develop their local implementation plan and then help them to execute the plan, as well as providing consultative support to facilities post-implementation [20]. Each LTC facility will be encouraged and supported to develop an implementation team, consisting of key stakeholders such as managers, nurse educators, front-line staff, and residents/family members.

Utilizing the Patient-Centered Outcomes Research Institute (PCORI) model of patient engagement [21–23], we recruited residents and family members to participate as members of the project team, through the formation of a Resident and Family Advisory committee (RFAC). In alignment with PCORI, members of the RFAC received training opportunities to more fully engage in this research project. The RFAC will be involved in several aspects of implementation and evaluation including oversight of the PRIHS intervention and ensuring that it reflects the needs of LTC residents and family members, direct involvement in designing both interview and focus group questions, acting as liaisons’ between the project and local RFAC groups at LTC facilities, providing leadership and oversight to a communications working group tasked with developing educational materials for residents and family members, and assisting in implementation by delivering their own experiences with having family members transferred from LTC to ED while helping to inform and engage the residents and family members from within the LTC facilities affected by this change in clinical practice.

### Intervention

**Fig. 2:**
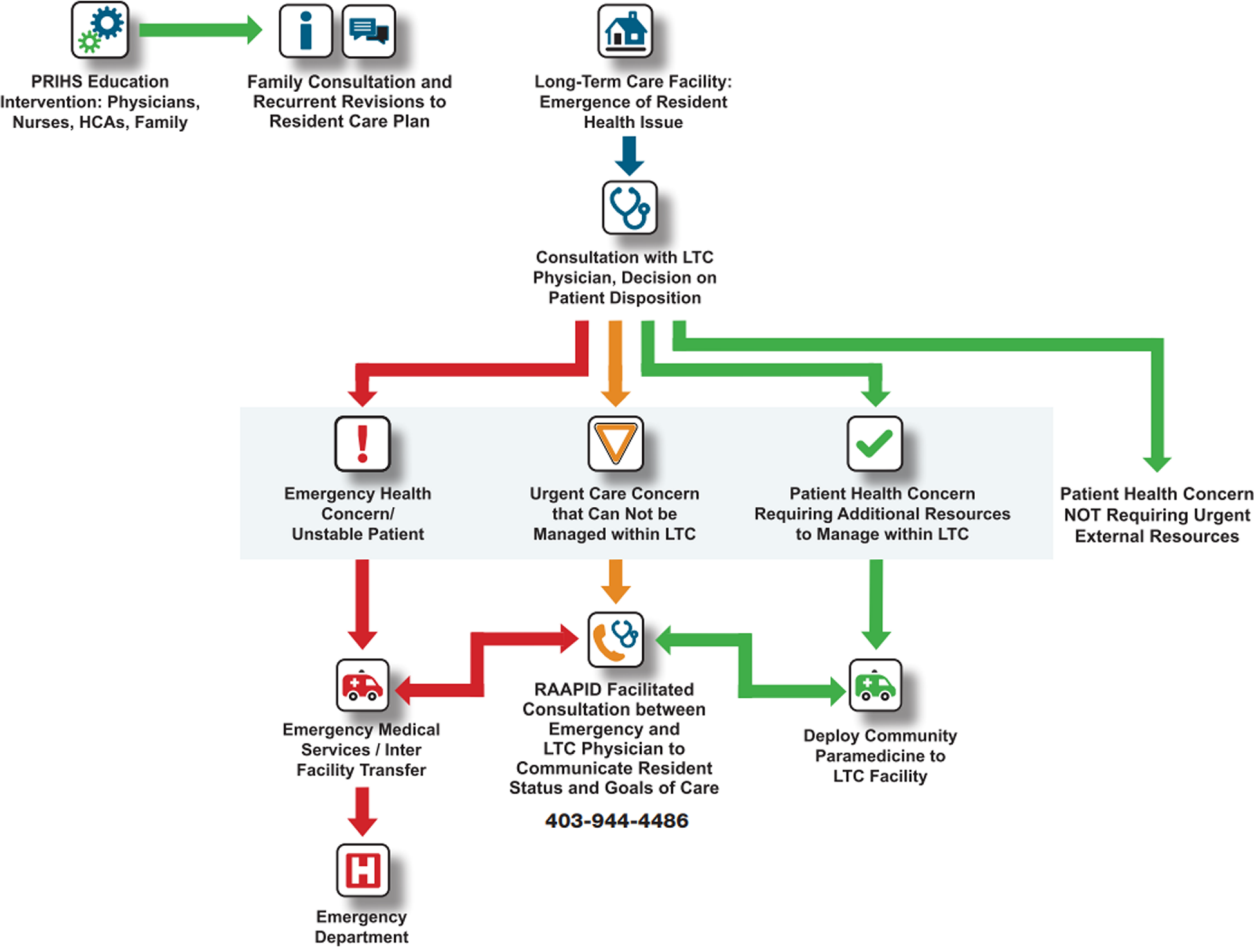
LTC to ED referral pathway

The intervention involves alterations in how current health care resources are utilized by LTC facilities. Specifically, we will implement a standardized LTC-ED care and referral pathway (Fig. 2), along with targeted INTERACT® (Interventions to Reduce Acute Care Transfers) tools [24]. INTERACT® is a quality improvement program that includes specific tools and pathways designed to improve the identification, evaluation, and communication around changes in LTC resident health status. This program was developed in the United States and the paper-based tools have been adapted for use in Canada. This project will focus specifically on two INTERACT® tools: The Stop-and-Watch Early Warning tool for HCAs and the Change in Condition file cards for nurses [24, 25].

HCAs’ familiarity with LTC residents put them in an ideal position to identify early changes in a resident’s status, using the Stop-and-Watch tool. Once notified by HCAs of a resident’s change in condition, nurses in LTC will be supported in making decisions using the Change in Condition cards and through early communication with the attending LTC or on-call physician. The goal is to support LTC in early identification and onsite management of changes in health status where appropriate, as well as to improve communication between LTC practitioners. Implementation of INTERACT® tools can result in a significant reduction in hospitalization rates for residents in LTC facilities [24, 25].

The LTC-ED care and referral pathway will serve as a resource for LTC facilities, when making management decisions for LTC residents experiencing an acute change. A call center that serves as a single point of contact for care providers, in order to facilitate care discussions and transfers between facilities (i.e., LTC and ED), will be used to link the LTC physician and nurse to an ED physician. The call centre is called RAAPID (Referral, Access, Advice, Placement, Information & Destination). RAAPID will support a discussion between health care providers regarding the best options for further assessment and care (e.g. ED transfer vs. community paramedicine visit) and help to minimize errors that can occur during transitions in care.

The pathway will also help to further optimize the use of community paramedicine by LTC facilities. The community paramedicine program provides primary and urgent health care to patients in the community, including LTC. The community paramedicine program also has procedures in place to provide facilitated transport to and from diagnostic imaging without an associated ED visit. Studies have shown that the provision of community paramedics within LTC significantly reduces transfers to the ED, improved outcomes, and improved resident and family experiences [12, 26–28].

Our evaluation of this change initiative will consider all six dimensions of quality outlined by the Alberta Quality Matrix for Health. Specifically, this evaluation will include an assessment of the impact on LTC-ED transfers, resident/family, health care provider experience, and health care system costs. A data visualization dashboard utilizing Tableau software and designed in partnership with AHS Clinical Analytics will capture facility specific administrative data on transfers. This audit feedback tool will be used as a control for sites to compare the rate of transfers before and after implementation. This data will be provided to the LTC facilities as part of semiannual audit-feedback reports.

### Evaluation plan

The evaluation framework will use a mixed-methods approach, where quantitative and qualitative data collection will occur using a concurrent dominant design. Mixed methods research involves collecting, analyzing, and interpreting quantitative and qualitative data in a single study that investigates the same underlying phenomenon [29]. Mixed methods research is useful for studying complex health issues such as motivations, attitudes, perceptions, beliefs, and values that underlie health behaviours within social, economic, organizational, and political contexts that cannot be captured by either quantitative or qualitative designs alone [30]. The primary outcome will be the change in the rate of transfers to ED from LTC facilities. Specifically, we will compare differences in the rate of transfers per 1000 resident days. This will be used to track the effectiveness of the intervention at the LTC sites (i.e., changes in the rate of transfer before and after the intervention). Data on all LTC-ED transfers are currently collected as part of administrative data by Alberta Health Service Analytics. Over the 30-month study period, our current rate of ED transfers would result in over 8000 LTC-ED transfers from patients within the 38 participating Calgary LTC facilities. After we account for the stepped-wedge design effects and plausible levels of over-dispersion, this will provide 80% power to detect a 15% reduction in the rate of LTC-ED transfers (primary outcome) [15, 31–33].

Secondary outcomes, using data obtained from administrative databases, will include the change in the rate of LTC admissions to hospital per 1000 resident days and the proportion of LTC residents cared for in their LTC facilities through community paramedicine post-pathway implementation. Consenting residents will also evaluate community paramedicine and ED care episodes by completing a modified version of the OPTICS (Older Persons Transition in Care Success; see Additional file 1) Success tool, within 2 days of a community paramedic visit or an emergency department visit that did not result in a hospital admission [31]. The OPTICS tool has been used and validated in previous studies [34, 37].

The appropriateness of the pathway triage decision will be assessed using a random

sample of up to 50 ED transfers and 50 community paramedicine visits. A standardized data collection form will be used by trained abstractors to extract key elements related to these episodes of care from medical records of consenting patients. A panel of physicians, including emergency physicians, geriatricians and physicians working within LTC, will review these completed abstraction forms to determine appropriateness of the triage decision, using the definitions of unnecessary and avoidable transitions developed in the EXACT (Examining Aged Care Transitions) study. This definition was developed through focus groups and interviews with LTC, EMS (emergency medical services) and ED staff and physicians, and then validated through a larger survey [35]. Additionally, the panel will determine if the treatment plan was concordant with residents’ wishes for treatment and goals of care designation.

Safety reviews of medical records will also be completed for up to 50 consenting patients who are treated by community paramedicine and subsequently transferred to ED within 72 h. A standardized form will be used by trained abstractors to extract key elements related to these episodes of care from medical records. These completed abstraction forms will then be reviewed by the physician panel. Specifically, we will use a previously developed six-point scale to determine the degree to which the ED transfer was the result of suboptimal care provided by community paramedicine [36].

Assessing the economic feasibility of this program is important to informing its scale and spread. A comparative cost-analysis will be undertaken, which includes hospitalizations, ED visits, ambulatory care, urgent care center, and physician and paramedic costs. This resource use is routinely captured by administrative data sets. Patient-level cost data will be provided by administrative data collected by the AHS. We will capture physician costs, hospital costs, as well as the cost of implementing our program.

To better explore if and why our intervention is effective, accessible, acceptable, and appropriate, we will conduct interviews and focus groups with patients and/or substitute decision-makers, families, and health care providers. A sample of consenting residents and/or family members will be interviewed using semi-structured interviews. We will interview a mix of residents transferred to ED and treated in LTC by community paramedics. Interviews were chosen in order to best accommodate for mild cognitive impairment and/or sensory impairment among participating residents. The interview questions were developed in consultation with resident and family advisors. The interviews will be 20–30 min in length to allow for an in-depth evaluation, without being too taxing for frail residents. Each interview will be recorded and transcribed verbatim, except for the exclusion of identifying information. Interviews will continue until there is saturation of themes, but it is expected that 15–30 interviews will be required in total [37, 38]. Qualitative data will be analyzed using thematic analysis and the software NVivo [39].

We will also conduct six semi-structured focus groups in total with involved health care providers, (i.e., with ED and LTC physicians, with LTC nurses and HCAs, with RAAPID staff and with community paramedics), in order to determine their experiences with the new LTC-ED care and referral pathway and early detection tools. Focus groups will facilitate a space in which participants are able to share their experiences and inform understandings of facilitators and barriers. Each focus group will last 60–90 min and include 6–12 participants. Focus groups will be recorded and transcribed verbatim, except for the exclusion of identifying information. Residents and/or family members and health practitioners will be interviewed separately.

In conjunction with AHS analytics we have developed an electronic dashboard specifically designed to support the evaluation of this project. The electronic dashboard was developed by AHS analytics in 2015 on the software Tableau. The dashboard captures monthly data on transfers from facilities. Only selected members of the research team can access the dashboard. Members of the project team (i.e., implementation coach) provide each facility with biannual reports of how the facilities are performing. The dashboard captures data on ED visits from each LTC enrolled in the study per 1000 resident days. RAAPID calls to LTC sites, visits to LTC sites by community paramedicine, and hospital admission rates per 1000 resident days for each facility enrolled in the study. This dashboard will be used as an audit-feedback tool for LTC facilities, as the initiative is scaled up and spread across the province. Additional administrative data captured on the dashboard will include transfer volume, ED arrival mode, primary diagnosis, CTAS score, and EMS events.

**Fig. 3:**
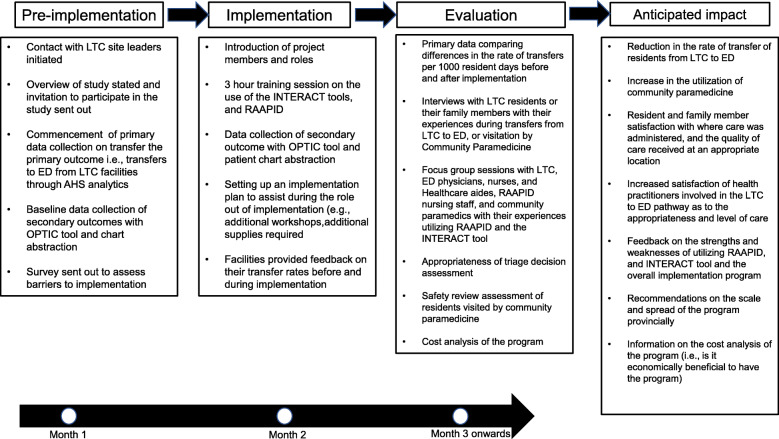
Logic model of the major components of the study

The major components of this study have been summarized in Fig. 3.

### Study participants

Administrative data related to LTC resident visits by a community paramedic(s) and/or emergency department visits will be included for the 38 participating facilities. Consenting LTC residents and family members will complete the OPTICS tool with between five to 12 participants being recruited per facility. Residents will typically be long-stay residents. Fifteen to thirty eligible and consenting LTC residents and family members will participate in interviews. Thirty-six to seventy-two health care providers working within the participating LTC facilities, community paramedicine, and emergency departments will be asked to participate in focus groups.

Inclusion criteria for recruitment of residents or their family members include

1. LTC Resident has been seen by a community paramedic(s) OR has returned directly from the emergency department within the past 2 days (i.e., 72 hours) and they were not admitted to the hospital.
2. The residents are not acutely confused.
3. Must have a RAI CPS (Cognitive Performance Scale) score of 2 or less completed within the past 6 months.
4. Do not have an enacted personal care directive (i.e., they are still able to make their own care decision)
5. LTC Residents > 65 years
6. If the LTC resident does not meet these criteria but there is a family member who is regularly visiting the LTC resident, the family member can be approached while visiting the LTC facility for permission to be contacted.

Focus groups will be conducted with physicians, nurses, community paramedics and health care aides, who are direct care staff in LTC facilities. Inclusion criteria for LTC and ED health practitioners.

1. Physicians, staff (i.e., nurses and health care aides), or community paramedics associated with the LTC or ED must be familiar with or must have utilized the RAAPID pathway during the duration of the study.
2. Nurses and health care aides must have used the INTERACT® and STOP and WATCH tools during the study to aid in their decision making.

### Analysis plan

#### Quantitative analysis

The rate of transfers to ED from LTC facilities will be expressed as the number of ED transfers per 1000 resident days within each three-month cluster period. For our primary outcome, we will use a negative-binomial generalized linear mixed effects model. This model will have random intercepts for sites, an indicator variable for treatment mode in each cluster at each period and time, and fixed effects for time [15]. This model will account for Poisson over-dispersion, as well as correlations among observations within sites [31–33]. As a secondary analysis, we will use an individual-level analysis to determine whether the impact of the intervention depends on available patient-level factors (such as sex) and in a multilevel mixed-effects model with patient-level and site level effects. Generalized linear mixed-effects models will be used to assess a time by intervention effect on other outcomes using the link functions required to meet model assumptions [40].

Descriptive statistics will be used to determine the proportion of LTC residents cared for in their LTC facilities by the community paramedicine program, using the new LTC-ED care and referral pathway. This will be expressed as the number of Mobile Integrated Health (MIH) community paramedic LTC visits per total RAAPID calls from LTC. Descriptive statistics will also be used to present data on the appropriateness and safety of the pathway triage decision. The median and interquartile range will be calculated for the modified OPTICS tool scores for each of the three groups: pre-intervention ED transfer, post-intervention ED transfers, and post-intervention community paramedicine visits. Time series analysis will be used to compare the different groups.

#### Qualitative analysis

Both the resident/family interviews and the health care provider focus groups will be evaluated using thematic content analysis [41]. Interviews and focus groups will be conducted by an individual with experience utilizing qualitative methods. For the focus group, a second individual will attend to take notes. Two analysts will then independently assess the qualitative data (notes and transcripts). Triangulation of the viewpoints of the two analysts will allow for a more complete understanding of the data, and exploration of how individual analyst’s preconceptions influence their interpretation [42, 43]. Reviewers will independently apply “codes” or labels to sections of the data within 10 transcripts (eight interviews; two focus groups). Initial codes will include the categories of the Health Quality Council of Alberta (HQCA) framework [44]. Other codes will be created by the analysts based on their understanding of the data. Analysts will meet together to compare codes. By separating cases where distinct issues have been categorized under one code (analysis) and bringing together distinct codes that speak to the same issue (synthesis), the analysts will create a finalized set of codes or “codebook.” The analysts will then code the remainder of the transcripts. Finally, analysts will review and discuss the meaning and context of the coded data with members of the research team, including patients and families, to validate their interpretations. Together, the analysts and research team members will develop overarching themes, which will be the final product of the qualitative analysis. Discrepancies in coding will be resolved by discussion or review of a third analyst if required.

Data integration of the quantitative and qualitative data will occur through merging. Merging is a data integration technique in which data collected through quantitative and qualitative methods are compared looking for convergence and divergence among the data [30]. Merging of data will provide a holistic understanding of how and why the intervention was effective. For example, by having the data on the change in the number of residents admitted to ED before and after the intervention for each facility coupled with qualitative data from health practitioners on the facilitators and barriers to implementation of RAAPID and the INTERACT tools, we will be able to provide LTC facility vendors with metrics on how well they performed on the intervention and recommendations to overcome site specific barriers (e.g., what was different about the facilities that performed well on the intervention compared to facilities where it was difficult to adopt the intervention). Furthermore, the qualitative data gathered from patients on their experiences either with a transfer to the ED or a visit by community paramedicine will enable us develop recommendations or policies that not only cater to the constraints of the health care system but more importantly, focus on patients’ outcomes and the experiences of the patient and/or their family members. This information will be important in informing the spread of this program to other health regions in Alberta and Canada.

#### Cost analysis

Comparative cost-analysis will be undertaken from the perspective of the publicly funded health care system. Health care costs will include hospitalizations, ED visits, ambulatory care, urgent care center, physician, and paramedic costs. Patient-level cost data will be provided by Data Integration, Measurement & Reporting (DIMR) at AHS. DIMR provides administrative data on costs as well as patient characteristics and diagnoses (ICD-10 codes). Physician costs are based on the fee for service physician billing to Alberta Health or, for those involved in alternative re-imbursement plans, shadow billing is used. Hospital costs are based on facility-specific costs per day and include direct costs and indirect costs such as diagnostic imaging, laboratory, housekeeping, administration etc. Monthly total costs will be calculated per patient by summing across all available cost data. A generalized linear model with random effects controlling for time and patient characteristics such as age and sex will be used to estimate the difference in costs with and without the program. Program costs, which are fully captured by our operational budget, will include wages, training, transportation, implementation resources, and IT costs. The difference in health care costs will be calculated and compared to program costs. These analyses will be used to evaluate the program’s return on investment. Costs will be reported at seven and 30 days.

### Ethical considerations

This study protocol has received approval from the University of Alberta Research Ethics Board (Pro 00090932) and the University of Calgary Conjoint Research Ethics Board (REB190106). The study received funding from Alberta Innovates Health Solutions and AHS. Informed consent will be sought from LTC residents or their family members to participate in the OPTICS tool survey, chart abstractions, and/or interviews. Health care practitioners involved with LTC, EMS or the ED will also consent to participate in focus group sessions.

## Discussion

Unnecessary transfers from LTC to the ED expose LTC residents to potential iatrogenic harms and increase their risk of cognitive and functional decline. This study will bring together current resources in a manner that will better coordinate and optimize their use, to improve the care received by LTC residents experiencing an acute change in status. Using a PCC approach to this study is essential. Patient centred care comprises of three major tenets: communication with patients, partnerships with health practitioners, and advocating for health promotion [45]. This project seeks improve the quality of care LTC residents receive by ensuring residents receive an appropriate level of care at the appropriate location by utilizing early detection tools for acute conditions in LTC residents and creating a framework for communication between LTC and ED physicians. This is anticipated to reduce the rate of transfer of residents from LTC to ED while increasing in the utilization of community paramedicine. Feedback from health practitioners on the utility of RAAPID, INTERACT® tool and barriers faced while implementing these changes to practice will be used to inform the scale and spread of this program to other health regions within the province. Lessons learned from the implementation of this study could potentially be used to inform future studies in LTC. With the ongoing COVID 19 pandemic and the heightened vulnerability of LTC residents to contracting COVID 19 this study will be crucial in ensuring residents receive the appropriate level of care at the appropriate location. This may reduce the strain on ED and free up staff and resources involved in combating the COVID 19 pandemic in hospitals. Furthermore, the use of early detection tools such as the INTERACT® tool may help in the early detection of COVID 19 in LTC facilities.

The intervention is expected to result in fewer transfers, as well as and better communication between LTC and ED practitioners when a transfer to ED is required, resulting in potential benefits for both LTC residents and the health care system.

## Supplementary Information

**Additional file 1.** OPTICS tool. Older Persons Transition in Care Success (OPTICS) tool (modified for use with ED transfers or community paramedicine visits). Questionnaire to be used to determine resident experiences with transfers to the ED or with visits from Community Paramedics.

**Additional file 2.** Focus group questionnaire. Focus group questionnaire for health practitioners. Sample of Focus group questionnaire to be administered to health practitioners based on the Theoretical domains framework.

**Additional file 3.** SPIRIT checklist. SPIRIT guidelines checklist for article.

**Additional file 4.** SPIRIT diagram. SPIRIT diagram for plan for study.

**Additional file 5.** Consent documents. Sample of Consent documents for LTC managers and Residents of their family members.

## Data Availability

Not applicable.

## Abbreviations

LTC: Long term Care Facility
ED: Emergency Departments
EMS: Emergency Medical Services
OPTICS: Older Persons Transition in Care Success
RAAPID: Referral, Access, Advice, Placement, Information & Destination
