# Supplementary material for: Improving care for residents in long term care facilities experiencing an acute change in health status
Source: BMC Health Serv Res. 2020 Nov 25;20:1075. doi: 10.1186/s12913-020-05919-7 (PMC7685962; doi:10.1186/s12913-020-05919-7)
Supplement: Supplementary file 1 — Additional file 1. OPTICS tool. Older Persons Transition in Care Success (OPTICS) tool (modified for use with ED transfers or community paramedicine visits). Questionnaire to be used to determine resident experiences with transfers to the ED or with visits from Community Paramedics. [file 12913_2020_5919_MOESM1_ESM.docx]

**Additional file 1: OPTICS tool**

Older Persons Transition in Care Success (OPTICS) tool (modified for use with ED transfers or community paramedicine visits)

Resident was: Transferred to the ED

OPTIC TOOL completed with:

LTC Resident

Family Member of LTC Resident

We are interested in **your thoughts** about your recent trip to the Emergency Department.

**SCREENING QUESTION:**

*Can you tell me the reason you were last transferred to the Emergency Department?*

*If the resident is clear about reason for transfer check € Yes and ask the following 3 questions.*

*If the resident is unclear about transfer check € No and proceed no further.*

YES

NO

*[Note: You can remind the resident of the reason for transfer and if they seem to recall you can try the OPTICS Tool with them]*

**Script for 1^st^ attempt:**

I will ask you a few questions about your transfer to the Emergency Department. Please answer on a scale of 1 to 5, with 1 being Very Good and 5 being Very Bad. [Use the laminated poster of the scale for the resident]

**1 2 3 4 5**

**Very Good Neutral Bad Very**

**Good Bad**

**Script for 2^nd^ attempt (only use if the resident seems unable to answer on the scale)**

I will read a few questions about your transfer to the Emergency Department. Please choose the face that best shows your answer: **[NOTE: hold up laminated poster]**

| 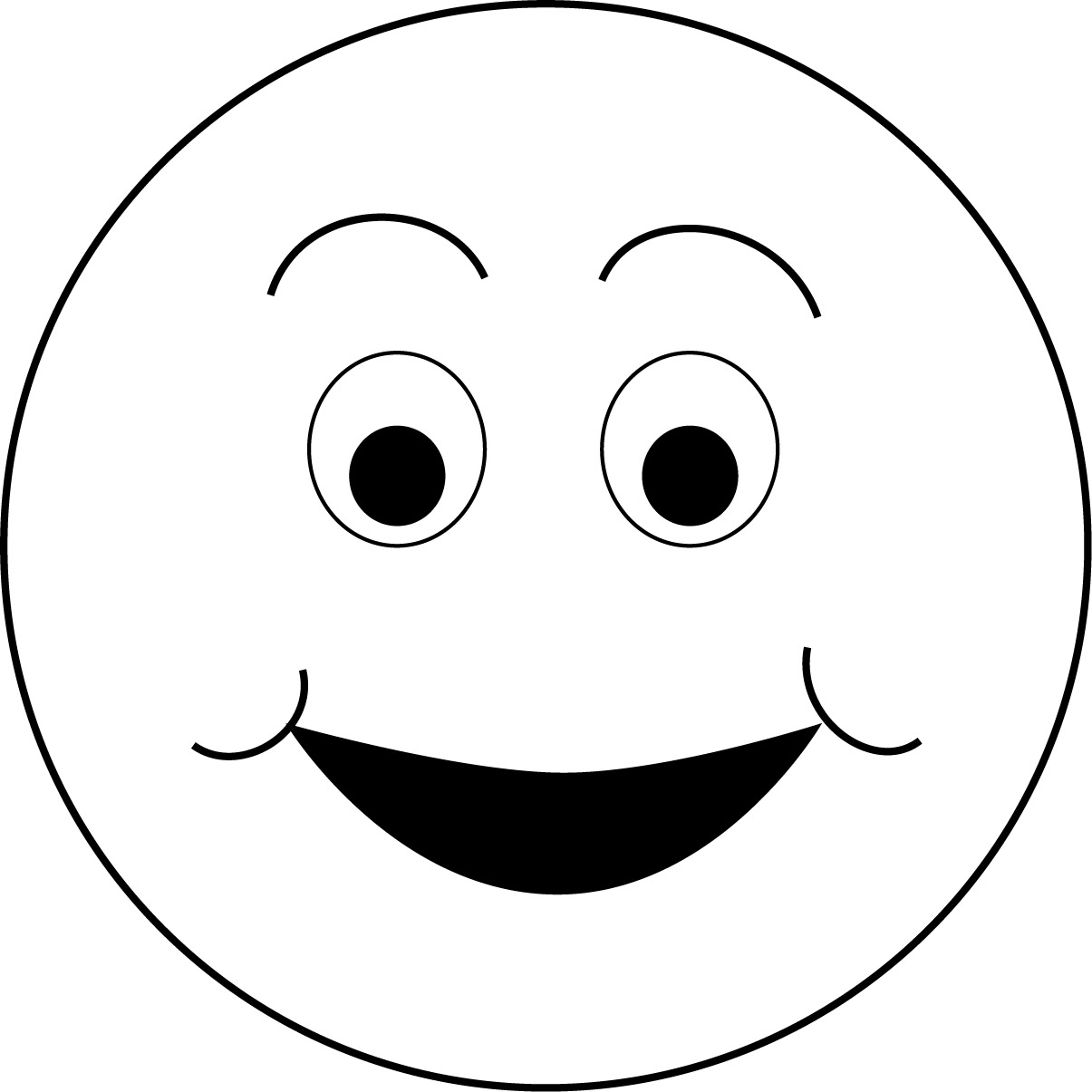 | 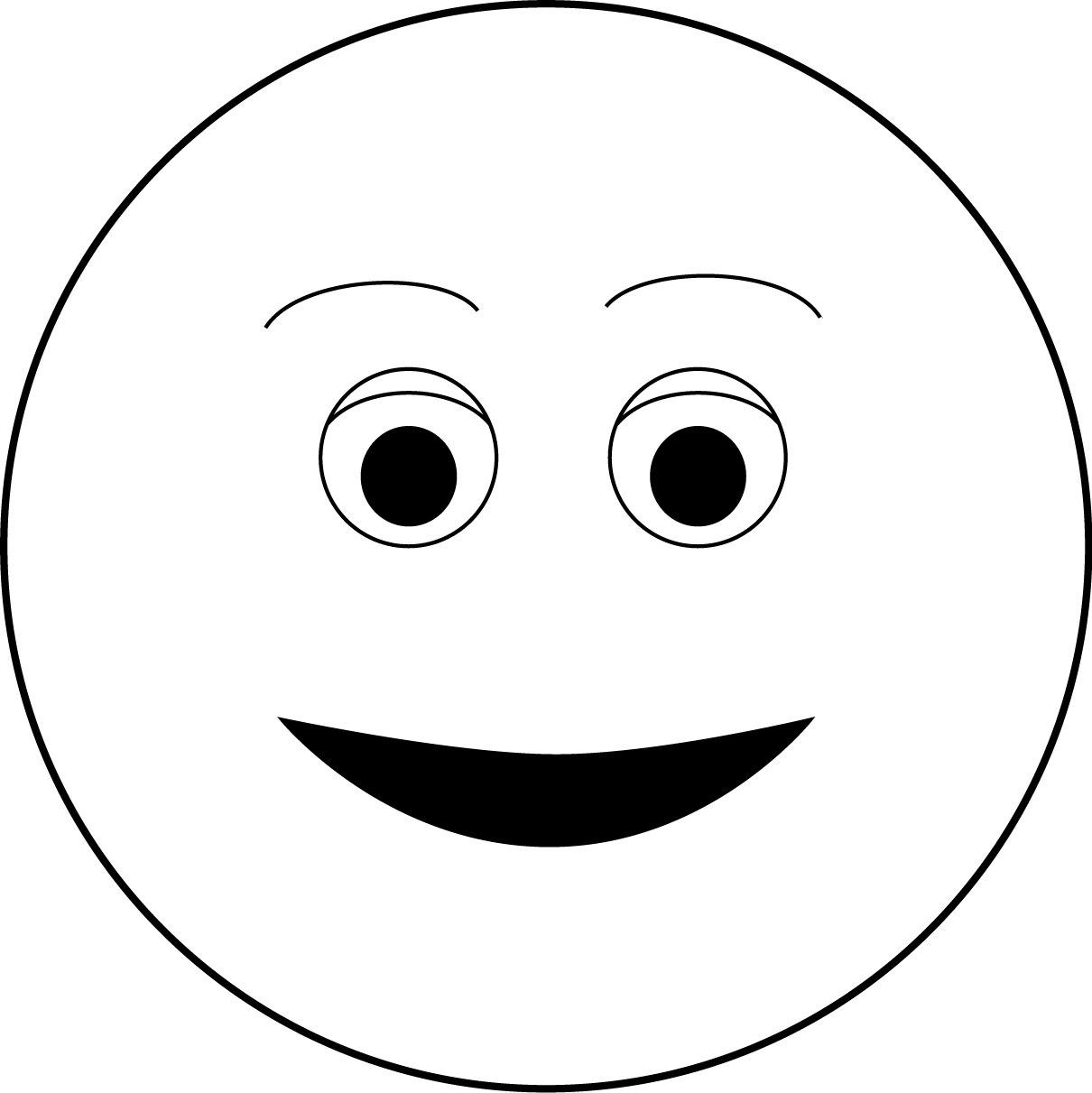 | 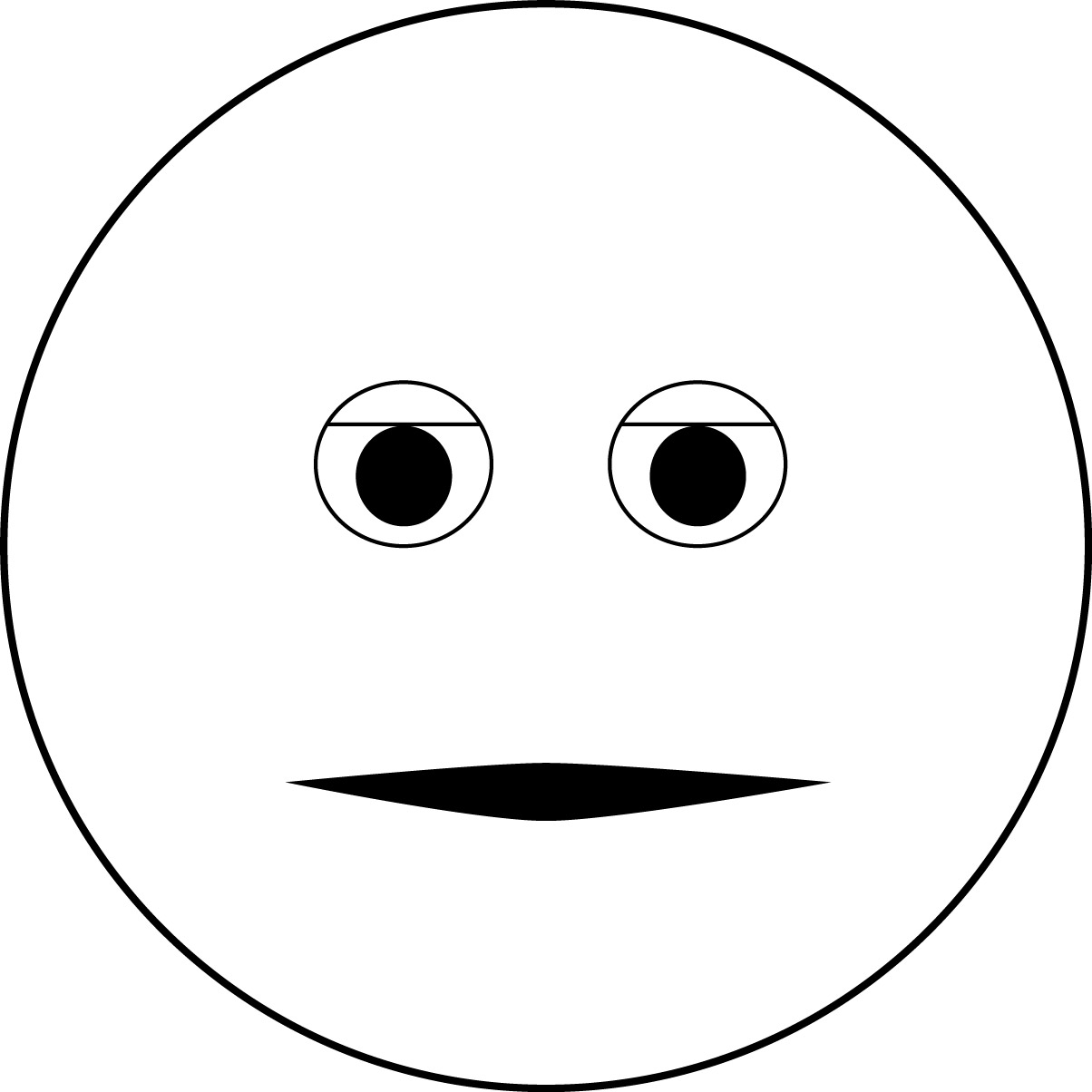 | 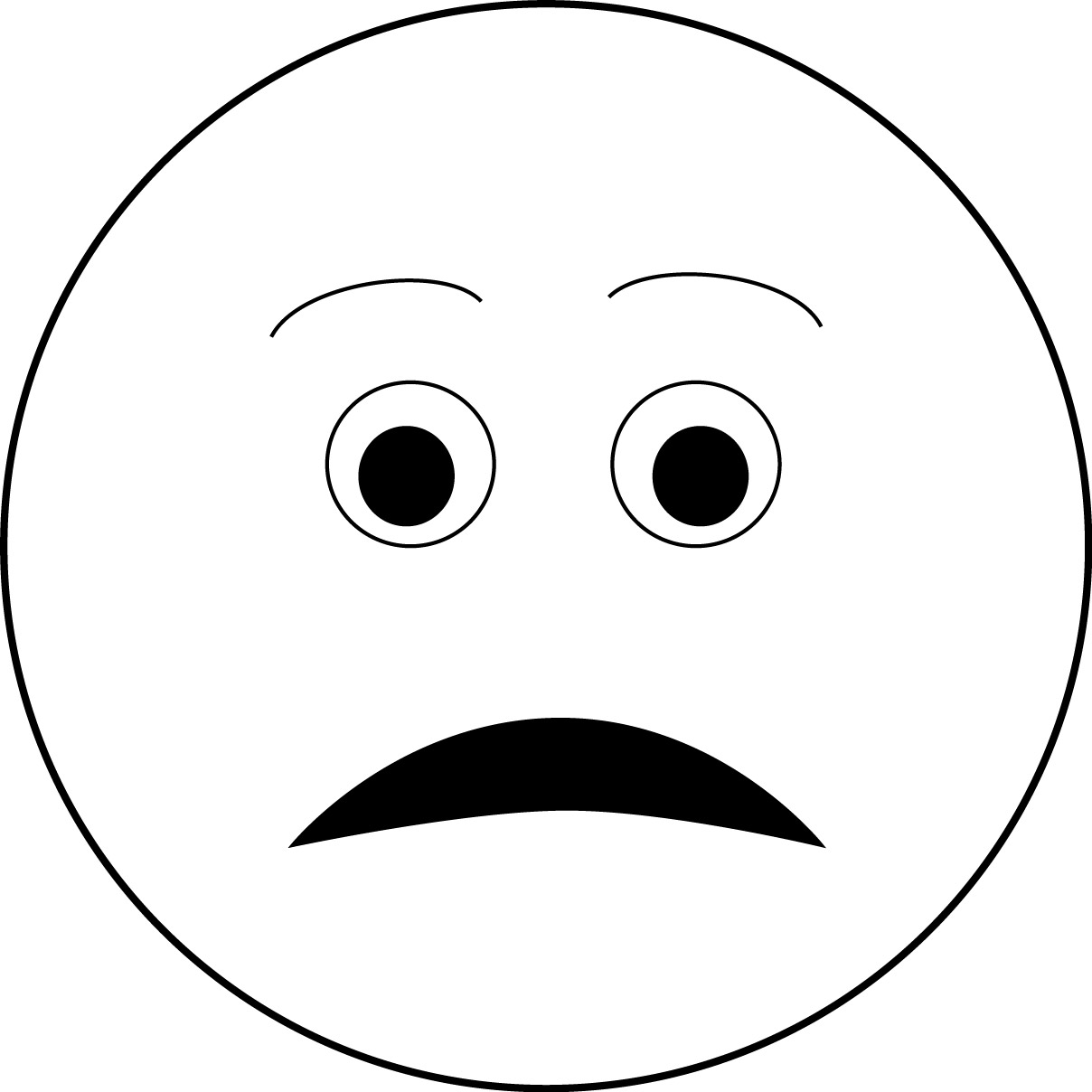 | 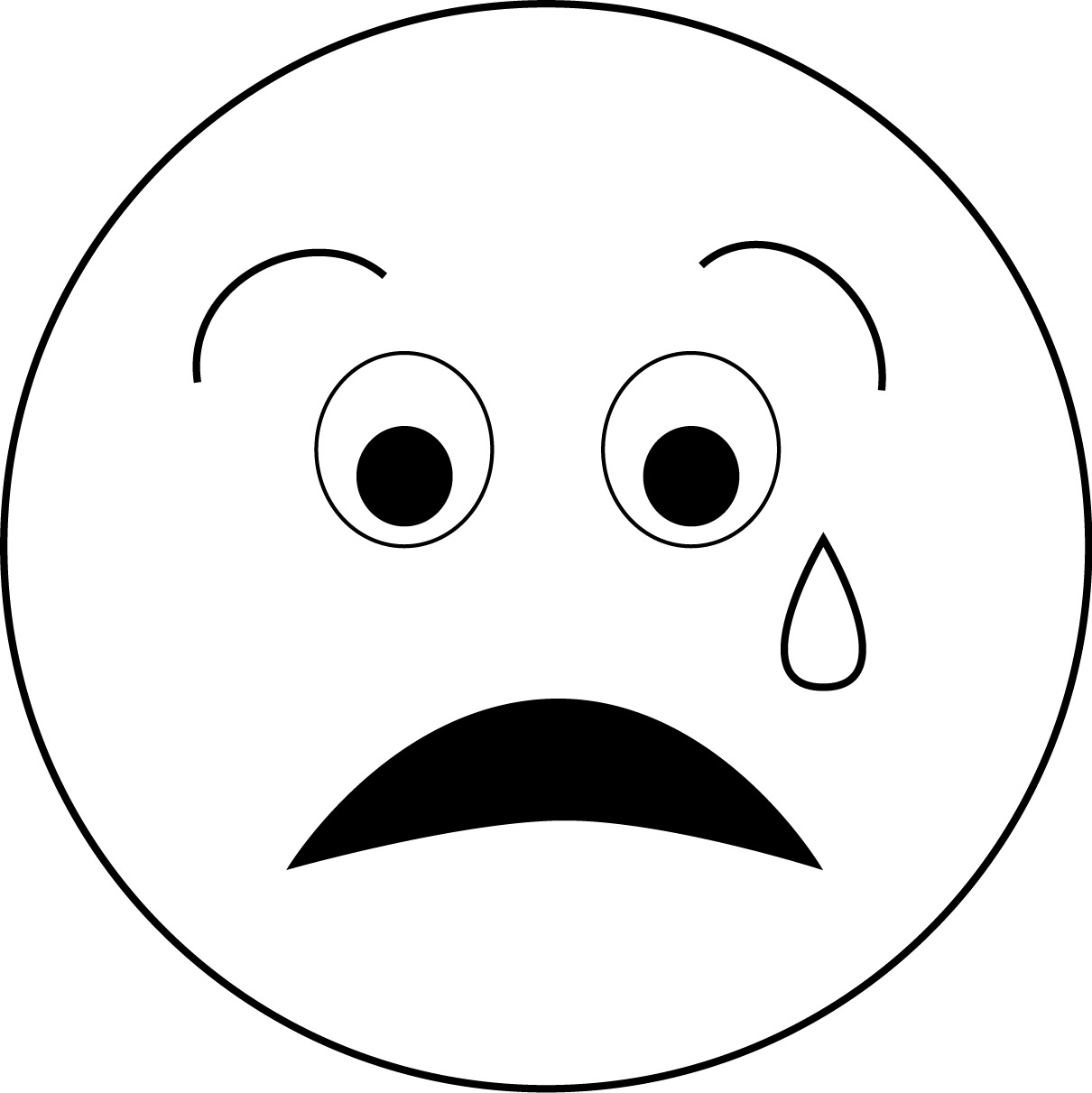 |
| --- | --- | --- | --- | --- |

[**Note:** When describing the responses for the faces use the following terms, Very good (Very Happy), Somewhat Good (Happy), Neutral, Somewhat not good (Unhappy), Not good (Very Unhappy)]

**1. How was the care you received in the ambulance?** [Prompt: Do you remember being in the ambulance?]

1 2 3 4 5 € Don’t recall

Very Good Neutral Bad Very

Good Bad

**2. How was the care you received in the emergency department?** [Prompt: Do you remember being in the emergency department?]

1 2 3 4 5 € Don’t recall

Very Good Neutral Bad Very

Good Bad

**3. Overall, how was your transfer?**

1 2 3 4 5 € Don’t recall

Very Good Neutral Bad Very

Good Bad

Would you have preferred to have Community Paramedics come to see you here at **[name of facility]** rather than go to the Emergency Department?

Yes

No

No preference

Field Notes (put in your observations about the interview and information that the resident shares about the transfer:
