# Supplementary material for: Improving care for residents in long term care facilities experiencing an acute change in health status
Source: BMC Health Serv Res. 2020 Nov 25;20:1075. doi: 10.1186/s12913-020-05919-7 (PMC7685962; doi:10.1186/s12913-020-05919-7)
Supplement: Supplementary file 2 — Additional file 2. Focus group questionnaire. Focus group questionnaire for health practitioners. Sample of Focus group questionnaire to be administered to health practitioners based on the Theoretical domains framework. [file 12913_2020_5919_MOESM2_ESM.docx]

**Additional file 2: Focus group questionnaire for health practitioners (LTC nurses)**

We are here to discuss the current project under way looking at improving the way we care for long term care residents in times of medical urgency

**Personal and professional experiences**

1. Tell us about your experience implementing the intervention?

*Prompts (to be asked if not discussed):*

- Have you heard of the INTERACT tools (i.e., STOP and WATCH/change in condition cards) and RAAPID?
- If so, could you explain what RAAPID is and how it is used?
- Could you walk us through the process of using the INTERACT tools and RAAPID?
- Tell us about your experience using the **INTERACT tools (i.e., Stop and Watch and change in condition cards)** and **RAAPID** to make decisions about the care of residents?
- How easy or difficult is it to use the **INTERACT tool** and **RAAPID**?
- What was the conventional way of caring to a resident who had an acute condition prior to the implementation of this program?
- What advantages do the intervention have over conventional models of care involving immediate transfer to EDs? (e.g. patient satisfaction/health outcomes).
- What disadvantages does this intervention have?

2. As LTC nurses, what was your initial perception of the intervention and its tools (i.e. INTERACT tools and RAAPID)?

*Prompts (to be asked if not discussed):*

- How did this perception influence the way you implemented the intervention?

3. What kinds of health situations are more appropriately handled by Community Paramedics (CPs) rather than EDs?

4. Have there been occasions when a CP assessment occurs and a decision is made to transfer the resident to the ED?

*Prompts (to be asked if not discussed):*

What accounted for the decision to transfer to ED?

Who made the decision to transfer?

5. What informs the choice between CPs and immediate ED transfers?

6. How well do immediate ED transfers serve the needs of LTC residents?

*Prompt (to be asked if not discussed):*

- Are the residents’ goals of care met by immediate ED transfers? (e.g. patient satisfaction/health outcomes).

7. How well do CPs serve the needs of residents?

*Prompt (to be asked if not discussed):*

- Are the residents’ goals of care met by first consulting with CPs? (e.g. patient satisfaction/health outcomes).

8. How has this intervention changed the way care is delivered to LTC residents?

*Prompts (to be asked if not discussed):*

- How easy or difficult was your work prior to the introduction of this intervention?
- Are you worried about making wrong decisions with the residents’ care?
- Do you feel confident implementing the intervention or using the **Stop and Watch** and **RAAPID** tools?

9. How well are LTC nurses supported to implement the intervention or use the **Stop and Watch** and **RAAPID** tools?

*Prompts (to be asked if not discussed):*

- Organizational/provincial/federal policies.
- Organizational cultures/procedures.
- Clarity or ambiguity of roles and responsibilities of the professionals/agencies involved. And how does clarity or ambiguity of roles impact the implementation process?
- Role of institutional culture, workplace environment, and leadership in the implementation process.

10. Do health workers have a sense of how things are going with this intervention or what difference its implementation makes?

**Implementation process**

11. What factors facilitate the implementation of the intervention?

*Prompts (to be asked if not discussed)*

- How much time does it take to make the call to RAAPID?
- How much time does it take to go through the checklists on the INTERACT tools i.e., the STOP and WATCH and the change in condition cards?
- Do you feel that your decision making regarding whether to treat a resident at your site (either at the LTC or ED) is helped or hindered using the INTERACT tools?

*Prompts (to be asked if not discussed):*

- What makes it easier or difficult to use the **INTERACT** and **RAAPID** tools?
- Doubts about the quality and source of evidence
- Personnel knowledge of the intervention.
- Organizational/provincial/federal policies.
- Organizational cultures/procedures.
- Challenges encountered implementing the intervention.
- Clarity or ambiguity of roles and responsibilities of the professionals/agencies involved. And how does clarity or ambiguity of roles impact the implementation process?

12. What factors hinder the implementation of the intervention? (Prompts: refer to question 11).

13. How often are residents and/or their families involved in decisions to receive care from CPs or transfer to EDs?

*Prompt (if not discussed)*

- *Who is the primary decision maker when deciding whether to transfer a resident to ED or to have them seen by CP onsite?*
- *In your experience as LTC nurses what factors go into those decisions?*

**Sense of effectiveness**

14. The objective of the intervention was to reduce avoidable transfers to ED, ensure appropriate use of healthcare resources, and by so doing improve the care of LTC residents. Has the intervention achieved this objective? If not, why?

*Prompts (to use if not discussed):*

- What factors facilitate or hinder the realization of this objective?
- Has the intervention improved the care of LTC residents?
- How effective are the INTERACT tools at aiding in the detection and recording of acute changes in LTC residents?
- Have you noticed a change in the frequency of visits by CPs to LTC facilities after RAAPID was implemented (or) have you noticed a change in the number of residents being transferred to ED?
- Are you optimistic that your colleagues are comfortable using the INTERACT tools and RAAPID?

15. Based on your experience, how confident are you that CPs can provide adequate care for LTC residents?

16. How would you describe the effectiveness of RAAPID at facilitating connections/communication between various healthcare professionals involved in implementing the intervention?

*Prompts (to use if not discussed):*

- Which groups of professionals use RAAPID to enhance their communication with each other? (e.g., between LTC and ED, or between CPs and ED).
- How does this communication influence the quality of care provided to LTC residents experiencing acute health problems?

17. What supports do you or your colleagues need to enhance implementation of the intervention, including the use of the **INTERACT tools** and **RAAPID**?

*Prompts (to use if not discussed):*

- Training needs/skills development
- Education needs
- Resources/resource reallocation (staffing, equipment, etc.)
- Information needs/communication processes
- Changes in institutional culture, workplace environment, and leadership in the implementation process.
- Incentives
