# Supplementary material for: Improving care for residents in long term care facilities experiencing an acute change in health status
Source: BMC Health Serv Res. 2020 Nov 25;20:1075. doi: 10.1186/s12913-020-05919-7 (PMC7685962; doi:10.1186/s12913-020-05919-7)
Supplement: Supplementary file 4 — Additional file 4. SPIRIT diagram. SPIRIT diagram for plan for study. [file 12913_2020_5919_MOESM4_ESM.docx]

**Additional file 4: SPIRIT Diagram**

|  | **STUDY PERIOD** | | | | | | | |
| --- | --- | --- | --- | --- | --- | --- | --- | --- |
|  | **Enrolment** | **Allocation** | **Post-allocation** | | | | | **Close-out** |
| **TIMEPOINT**** | ***-t_1_*** | **0** | ***t_1_*** | ***t_2_*** | ***t_3_*** | ***t_4_*** | ***etc.*** | ***t_x_*** |
| **ENROLMENT:** |  |  |  |  |  |  |  |  |
| **Eligibility screen** | X |  |  |  |  |  |  |  |
| **Informed consent** | X |  |  |  |  |  |  |  |
| ***Introductory meeting*** | X |  |  |  |  |  |  |  |
| **Pre-implementation** |  | X |  |  |  |  |  |  |
| **INTERVENTIONS:** |  |  |  |  |  |  |  |  |
| ***Introductory meeting*** |  |  |  |  |  |  |  |  |
| ***Implementation of STOP and WATCH and INTERACT tool*** |  |  | X |  | X |  |  |  |
| **ASSESSMENTS:** |  |  |  |  |  |  |  |  |
| ***Rate change in rate of transfer from ED to LTC*** | X | X | X | X | X | X | X | X |
| ***OPTIC tool and Chart Abstraction*** |  | X | X | X | X | X | X | X |
| ***Comparative cost analysis*** |  | X | X | X | X | X | X | X |

*Recommended content can be displayed using various schematic formats. See SPIRIT 2013 Explanation and Elaboration for examples from protocols.

**List specific timepoints in this row.
