# Supplementary material for: Improving care for residents in long term care facilities experiencing an acute change in health status
Source: BMC Health Serv Res. 2020 Nov 25;20:1075. doi: 10.1186/s12913-020-05919-7 (PMC7685962; doi:10.1186/s12913-020-05919-7)
Supplement: Supplementary file 5 — Additional file 5. Consent documents. Sample of Consent documents for LTC managers and Residents of their family members. [file 12913_2020_5919_MOESM5_ESM.docx]

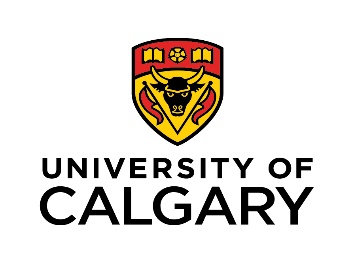

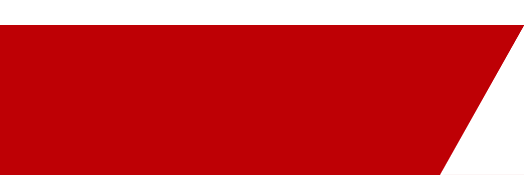


| **Improving Acute Care for Long-Term Care Residents:**  **A Better Way to Care for the Frail Elderly in Times of Medical Urgency**  **CONSENT TO CONTACT FORM** |
| --- |

**INSTRUCTIONS FOR LTC MANAGER OR DESIGNATE:**

If a LTC Resident has been **seen by a community paramedic(s**) OR has **returned directly from the emergency department** within the **past 2 days** (i.e. they were not admitted to the hospital)

AND

1. are **not acutely confused**
2. have **a RAI CPS** (Cognitive Performance Scale) **score of 2 or less** (completed within the past 6 months)
3. **do not** have an **enacted Power of Attorney** ( i.e. they are still able to make their own care decision)

Please approach the resident with this request to be contacted.

Alternatively, if the LTC resident does not meet these criteria but there is a family member who is regularly in visiting the LTC resident, the **family member can be approached while visiting the LTC facility for permission to be contacted**.

**CONSENT-TO- CONTACT SCRIPT FOR LTC MANAGER OR DESIGNATE** (to be read to the LTC Resident):

Hello my name is _______________________. I work at **[name of LTC facility]** as **[provide your role]**. Our facility is involved in the **Improving Acute Care for Long-Term Care Residents study**. This study involves carrying out and assessing a new care pathway for use by LTC facilities seeking transfer of a LTC resident to the emergency department.

I am here to ask your permission to provide your contact information to the Study Team. If you agree, they will contact you and ask you some questions about your (your family member’s) recent trip to the Emergency Department/care you ( your family member) recently received by the community paramedics within our long-term care facility.

You do not have to agree to provide your contact information. You do not have to participate. The benefits to you if you take part is that this may help to improve the quality of care of long-term care residents when they become sick. There are no risks if you participate. If you have questions about your rights as a research participant or the conduct of the study, you may contact the University of Calgary Conjoint Health Research Ethics Board at 403-220-7990. It will not affect your (your family member’s) access to medical care now or in the future. Are you willing?

**Instructions after sharing the above information with the LTC resident:** If the resident (family member) agrees to take part, please call one of the people listed below and provide the resident’s (family member’s) name and location (phone number).


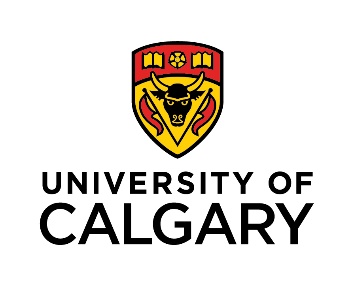

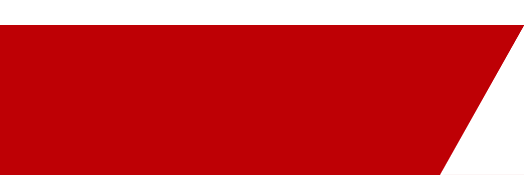


| **Improving Acute Care for Long-Term Care Residents:**  **A Better Way to Care for the Frail Elderly in Times of Medical Urgency**  **Consent to Complete OPTIC (Older Persons Transition in Care) TOOL** |
| --- |

**SPONSORS:** Alberta Innovates Health Solutions and Alberta Health Services

**PRINICPAL INVESTIGATOR:** Dr. Jayna Holroyd-Leduc

**OPERATIONAL LEAD:** Heather Hair

RESEARCH COLLABORATORS: Eddy Lang, Greta Cummings, Eldon Spackman, Patrick McLane and Peter Faris, Neil Collins, Nancy Zuzic, Michael Szava-Kovats, James Andruchow, Petra Bourqui, Vivian Ewa, Patrick Quail, and MaryJane Shankel

This consent form is only part of the process of informed consent. It should give you the basic idea of what the research is about and what your participation will involve. If you would like more details about something mentioned here, or information not included here, please ask. Take the time to read this carefully and to understand any accompanying information. You will receive a copy of this form.

**BACKGROUND**

- 15,000 Albertans live in Long Term Care (LTC) facilities,
- Every day 28 LTC residents are transferred to an Emergency Department (ED).
- Many of these changes in a resident’s condition could be addressed at the LTC facility if appropriate supports were available.
- Transfer to the ED and hospital are associated with increased risk of:
  - infections
  - falls and functional decline
  - delirium (confusion)
- When ED visits are appropriate, poor communication between the ED and LTC facility can lead to:
  - poor care and poor use of resources
  - patient and family dissatisfaction
  - poor outcomes

**WHAT IS THE PURPOSE OF THE STUDY?**

This study involves carrying out and assessing a new care pathway for use by LTC facilities seeking transfer of a LTC resident to the ED. This pathway will link the LTC doctor and nurse to an ED doctor, in order to help determine the best options for care (e.g. ED transfer vs. community paramedic visit).

**WHAT WOULD I HAVE TO DO?**

We are asking you to evaluate your (your family member’s) experience with your ( your family member’s) recent transfer to the ED (emergency department) or your ( your family member’s) recent assessment by a community paramedic(s) at your LTC facility. This will involve answering questions based on the OPTIC (Older Persons Transition in Care) Success tool. This will involve answering four (4) brief questions.

**WHAT ARE THE RISKS?**

There are no known harms related with completing the OPTIC tool. If a question is not relevant to you or you feel uncomfortable answering just let the assessor know. You may decline to answer any questions or withdraw from the study at any time.

**WILL I BENEFIT IF I TAKE PART?**

The information learned from this study will be used to improve the care provided to LTC residents when they become sick.

**DO I HAVE TO PARTICIPATE?**

Taking part in the study is voluntary and you may leave the study at any time. Please notify the assessor at any time should you wish to withdraw. The study team can also withdraw you from the study. In either case, your data will be removed as able and will not be used in the analysis.

**WILL I BE PAID FOR PARTICIPATING, OR DO I HAVE TO PAY FOR ANYTHING?**

There are no costs for taking part in this study. You will not be paid for taking part in this study.

**WILL MY RECORDS BE KEPT PRIVATE?**

The study team will have access to the data collected and the identity of participants. Data collected by the OPTIC tool will be anonymized prior to data analysis (i.e. identifying data removed), and the identity of participants will not be disclosed to anyone outside of the study team. Authorized representatives from the University of Calgary and the Conjoint Health Research Ethics Board may look at your identifiable medical/clinical study records held at Foothills Medical Centre and on RedCap secure online database for quality assurance purposes.

**SIGNATURES**

Your signature on this form indicates that you have understood to your satisfaction the information regarding your participation in the research project and agree to take part as a participant. In no way does this waive your legal rights nor release the investigators or involved institutions from their legal and professional responsibilities. You are free to withdraw from the study at any time without jeopardizing your health care. If you have further questions concerning matters related to this research, please contact:

**Dr. Jayna Holroyd-Leduc (403) 944-1771**

If you have any questions concerning your rights as a possible participant in this research, please contact the Chair, Conjoint Health Research Ethics Board, University of Calgary at 403-220-7990.

| Participant’s Name |  | Signature and Date |
| --- | --- | --- |
|  |  |  |
| Investigator/Delegate’s Name |  | Signature and Date |
|  |  |  |
| Witness’ Name |  | Signature and Date |
|  |  |  |

The University of Calgary Conjoint Health Research Ethics Board has approved this research study. A copy of this consent form has been given to you to keep for your records and reference.

**FACULTY OF MEDICINE, Department of Medicine, Division of Geriatric Medicine**

Dr. Jayna Holroyd-Leduc, **Foothills Medical Centre, 1403 29 Street NW, Calgary, AB, T2N 2T9, P: 403.944.1771**
